# ZFP36 Alleviates MASLD Through Facilitating TEAD4 mRNA Degradation After Sleeve Gastrectomy

**DOI:** 10.3390/ijms27093736

**Published:** 2026-04-22

**Authors:** Zhiyuan Tang, Min Sun, Junqiang Chen, Bowen Shi, Tianming Yu, Sanyuan Hu

**Affiliations:** Cheeloo College of Medicine, Shandong University, Jinan 250012, China; tangzy000108@163.com (Z.T.); sun20180427@163.com (M.S.); 15610118267@163.com (J.C.); bowenshi1997@outlook.com (B.S.); ikemind@163.com (T.Y.)

**Keywords:** MASLD, sleeve gastrectomy, ZFP36, TEAD4, hippo signaling pathway

## Abstract

RNA degradation plays a vital role in post-transcriptional regulation of gene expression. RNA stability is changed in the pathogenesis of metabolic dysfunction-associated steatotic liver disease (MASLD), but its role and underlying mechanisms in sleeve gastrectomy (SG) effectively remodeling hepatocytes and improving MASLD is unclear. A high-fat diet-induced MASLD model for SG and a hepatocyte-specific Zfp36 knockdown mouse model were established to evaluate the role of zinc finger protein 36 (ZFP36) in MASLD. The expression of ZFP36 and TEA domain transcription factor 4 (TEAD4) was examined in liver tissue samples from MASLD patients. Hepatic ZFP36 expression is downregulated in MASLD but is restored following SG. Hepatocyte-specific Zfp36 knockdown exacerbates high-fat diet-induced liver injury and impairs the therapeutic effect of SG on hepatic steatosis. Mechanistically, ZFP36 binds to TEAD4 mRNA to promote its degradation, thereby modulating the Hippo pathway. Inhibition of TEAD4 transcriptional activity reverses the aggravated MASLD phenotype caused by Zfp36 knockdown. In liver biopsy samples from MASLD patients, ZFP36 expression correlates negatively with TEAD4 expression. Collectively, these findings identify SG-induced upregulation of ZFP36 as a critical mechanism for alleviating MASLD through suppression of TEAD4.

## 1. Introduction

Metabolic dysfunction-associated steatotic liver disease (MASLD) has emerged as the most prevalent chronic liver disease worldwide [1]. It encompasses a spectrum ranging from simple hepatic steatosis to steatohepatitis, which can progress to cirrhosis, liver failure, and even hepatocellular carcinoma [2,3]. MASLD is a common comorbidity of obesity and is frequently associated with various metabolic abnormalities, such as insulin resistance and dyslipidemia [2]. Bariatric surgery, particularly sleeve gastrectomy (SG), is widely employed to treat obesity and its complications [4]. SG has demonstrated significant therapeutic efficacy against MASLD [5,6]. However, the underlying molecular mechanisms remain incompletely understood.

Post-transcriptional regulation of RNA plays a crucial role in MASLD progression [7,8]. For instance, death-associated protein kinase-related apoptosis-inducing kinase-2 (DRAK2) competitively binds to and inhibits the phosphorylation of serine- and arginine-rich splicing factor 6 (SRSF6), thereby impeding its nuclear import. This leads to the accumulation of aberrant RNA species, mitochondrial dysfunction, and ultimately promotes MASLD development [9]. Additionally, methyltransferase 3 (METTL3) mediates m^6^A modification of Rubicon mRNA, enhancing its stability and expression, which in turn suppresses autophagosome–lysosome fusion, impairs lipid droplet clearance, and contributes to hepatic lipid accumulation [10]. Also, it has been well documented that RNA-binding proteins modulate the progression of MASLD by regulating gene expression through controlling mRNA stability [11]. In activated hepatic stellate cells (HSCs), elevated CUGBP Elav-like family member 1 (CUGBP1) expression upregulates interferon gamma (IFN-γ) production by binding to and stabilizing its mRNA, which in turn activates the transforming growth factor beta (TGF-β) signaling pathway, thereby promoting liver fibrosis [12]. In contrast, in hepatocytes, ELAV-like RNA-binding protein 1 (HuR) binds to the 3′-untranslated regions (3′-UTR) of phosphatase and tensin homolog (*PTEN*) mRNA, enhances its stability, and thereby modulates lipid and glucose metabolism, ultimately inhibiting the progression of MASLD [13]. Investigating and elucidating the post-transcriptional regulatory networks, particularly exploring the interactions between RNA-binding proteins and RNAs, holds the potential to identify novel therapeutic targets for MASLD. As an RNA-binding protein, zinc finger protein (ZFP36) has been shown to bind AU-rich elements (ARE) in the 3’-UTR of target mRNAs via its zinc finger domain. It then recruits the CCR4-NOT complex through its NOT1-binding domain (NOT1BD) to deadenylate mRNAs, leading to their degradation by exonucleases [14]. In vascular smooth muscle cells, ZFP36 suppresses regulator of G protein signaling 2 (RGS2) expression, regulates intracellular calcium concentration, and thereby modulates smooth muscle contraction and blood pressure homeostasis [15]. In hepatic stellate cells, ZFP36 inhibits autophagy by degrading autophagy-related 16-like 1 (ATG16L1) mRNA, consequently suppressing ferroptosis [16]. Nevertheless, whether ZFP36 in hepatocytes contributes to the amelioration of MASLD by SG remains unclear.

The Hippo signaling pathway is a key regulator of hepatocyte proliferation and regeneration, and has also been implicated in MASLD pathogenesis [17,18,19]. Yes-associated protein (YAP) and WW domain-containing transcription regulator 1 (TAZ) are central effectors of this pathway. Their phosphorylation leads to cytoplasmic retention and degradation, whereas dephosphorylation promotes nuclear translocation, where they bind to transcription factors of the TEAD family to initiate downstream gene expression [20]. While TEA domain transcription factor 1 (TEAD1) has been found to activate lipid synthesis-related genes in the liver, the potential role of other TEAD family members, particularly TEA domain transcription factor 4 (TEAD4), in MASLD progression has not been reported [21].

In this study, we found that ZFP36 expression is downregulated in MASLD but upregulated following SG treatment. Through RIP-seq, we identified the Hippo signaling pathway as the following target of ZFP36 in modulating hepatic lipid metabolism. Mechanically, TEAD4 mRNA is a core responding molecule in the Hippo signaling pathway. Hepatocyte-specific knockdown of ZFP36 inhibited the degradation of TEAD4 mRNA, thereby promoting its expression and subsequently upregulating genes involved in lipid synthesis and fibrosis, which exacerbated MASLD. Collectively, these findings suggest that ZFP36 may serve as a potential therapeutic target for MASLD.

## 2. Results

### 2.1. ZFP36 Expression Is Downregulated in MASLD Liver Tissues

It is notable that some RNA-binding proteins are immediate-early genes that can quickly regulate mRNA stability and translation in response to a stimulus and metabolic changes [22]. To investigate the role of post-transcriptional RNA regulation in the development and progression of MASLD, we analyzed transcriptomic data from GSE246211. GSEA indicated that the RNA degradation pathway was significantly downregulated in liver tissues of MASLD mice induced by a 32-week high-fat diet (Figure S1A). Identifying specific RNAs and mediating their degradation may be a key step in suppressing MASLD progression. Zinc finger protein 36 (ZFP36) is one of the key proteins responsible for recognizing and promoting mRNA degradation.

**Figure 1:**
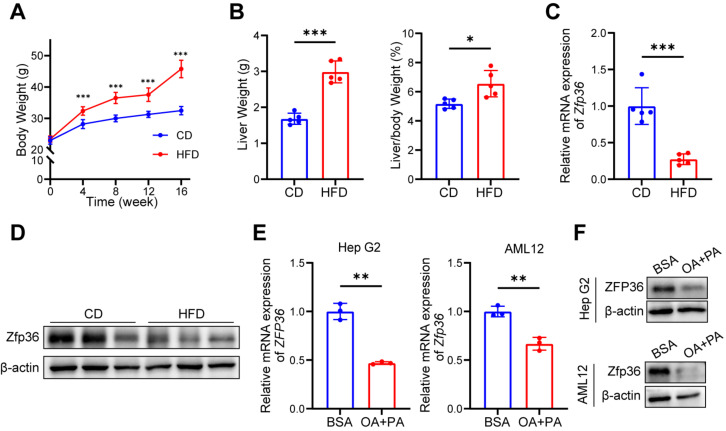
ZFP36 expression is downregulated in MASLD liver tissue. (**A**–**D**) Six-week-old male mice fed with chow diet (CD) (*n* = 5) or high-fat diet (HFD) (n = 5) for 16 weeks. (**A**) Mouse body weight, every 4 weeks. (**B**) Liver weight and liver-to-body weight ratio. (**C**) mRNA level of Zfp36 in mouse liver. (**D**) Protein level of Zfp36 in mouse liver. (**E**) mRNA level and (**F**) protein level of *ZFP36* in Hep G2 or AML12 cells treated with oleic acid (OA, 0.2 mM) and palmitic acid (PA, 0.1 mM); control group was treated with BSA. (* *p* < 0.05, ** *p* < 0.01, *** *p* < 0.001).

We established an MAFLD model by feeding mice with a high-fat diet (HFD) for 16 weeks. Compared with chow diet mice, the HFD mice exhibited significantly increased body weight, liver weight, liver-to-body weight ratio, and white adipose tissue mass (Figure 1A,B and Figure S1B). The markers of liver injury, alanine aminotransferase (ALT) and aspartate aminotransferase (AST), as well as lipid metabolites, triglycerides (TG), and total cholesterol (TC), were elevated both in peripheral blood and liver tissues, indicating hepatocyte damage and disrupted lipid metabolism induced by the high-fat diet (Figure S1C–F). Intraperitoneal glucose tolerance test (IPGTT) and insulin tolerance test (ITT) assays revealed impaired glucose tolerance and insulin tolerance in high-fat diet mice (Figure S1G,H). Histopathological examination further demonstrated increased lipid deposition in the liver tissues of these mice (Figure S1I). The expression of genes associated with liver fibrosis (*Ihh*) and fatty acid/cholesterol synthesis (*Srebf1*, *Fasn*, *Hmgcr*) was markedly upregulated in HFD mice liver samples (Figure S1J). We examined ZFP36 expression in mouse liver tissues and found that both RNA and protein levels of ZFP36 were significantly reduced in high-fat-diet mice, consistent with the database analysis results (Figure 1C,D). In vitro, lipid accumulation was induced in HepG2 and AML12 cells with oleic acid and palmitic acid addition. The results showed that oleic acid and palmitic acid treatment led to decreased ZFP36 expression (Figure 1E,F). Taken together, these data highlight a potential role of ZFP36 in the progression of MASLD.

### 2.2. ZFP36 Expression Is Upregulated in Liver Tissues After SG Surgery

**Figure 2:**
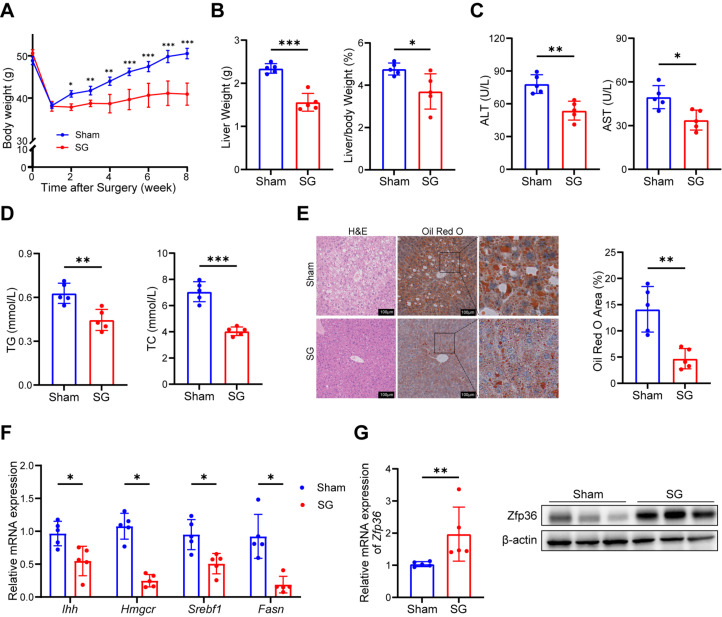
ZFP36 expression is upregulated in mouse liver tissue after sleeve gastrectomy. (**A**–**G**) HFD mice underwent sleeve gastrectomy (SG) (*n* = 5) or sham treatment (*n* = 5). (**A**) Mouse body weight after surgery. (**B**) Liver weight and liver-to-body weight ratio. (**C**) Serum ALT and AST content. (**D**) Serum triglyceride (TG) and total cholesterol (TC) levels. (**E**) H&E and Oil Red O staining of liver section, with Oil Red O positive area value. Scale bar, 100 μm. Magnification: 40×. (**F**) mRNA level of *Ihh*, *Hmgcr*, *Srebf1* and *Fasn* in mouse liver. (**G**) mRNA and protein levels of Zfp36 in mouse liver. (* *p* < 0.05, ** *p* < 0.01, *** *p* < 0.001).

Sleeve gastrectomy (SG) is one of the effective treatments for MASLD. To determine whether ZFP36 was changed in SG ameliorating MASLD, we performed SG or sham surgery on obese mice fed a high-fat diet. At one week post-surgery, body weight decreased in both groups, likely due to surgical stress and a transient reduction in food intake postoperatively. Despite continued high-fat diet feeding, the SG group showed a more pronounced reduction in body weight compared to the sham group (Figure 2A). By 8 weeks after surgery, SG mice exhibited significantly lower liver weight, liver-to-body weight ratio, and white adipose tissue mass (Figure 2B and Figure S2A). Markers of liver injury (ALT and AST) and lipid levels (TG and TC) in both peripheral blood and liver tissues were markedly reduced, indicating that SG surgery significantly alleviated liver injury and improved lipid metabolism disorders (Figure 2C,D and Figure S2B,C). Intraperitoneal glucose and insulin tolerance tests (IPGTT and ITT) showed that SG mice had lower fasting blood glucose levels and significantly improved glucose tolerance and insulin sensitivity (Figure S2D,E). H&E staining of liver tissue section revealed a notable reduction in hepatocellular swelling and vacuolization in SG mice, and Oil Red O staining further confirmed a significant decrease in hepatic lipid deposition (Figure 2E). Furthermore, the expression of Indian hedgehog protein (*Ihh*), sterol regulatory element-binding protein 1 (*Srebf1*), fatty acid synthase (*Fasn*) and 3-hydroxy-3-methylglutaryl-coenzyme A reductase *(Hmgcr*) was significantly downregulated in liver tissues, indicating suppressed fibrosis and reduced lipid synthesis (Figure 2F).

We extracted RNA and protein from mouse liver tissues. qPCR and Western blot analyses revealed that ZFP36 expression was significantly upregulated in the liver tissues of SG mice (Figure 2G). In summary, SG ameliorated MASLD in mice, and ZFP36 might play a vital role in its effect.

### 2.3. Hepatocellular ZFP36 Alleviates MAFLD

**Figure 3:**
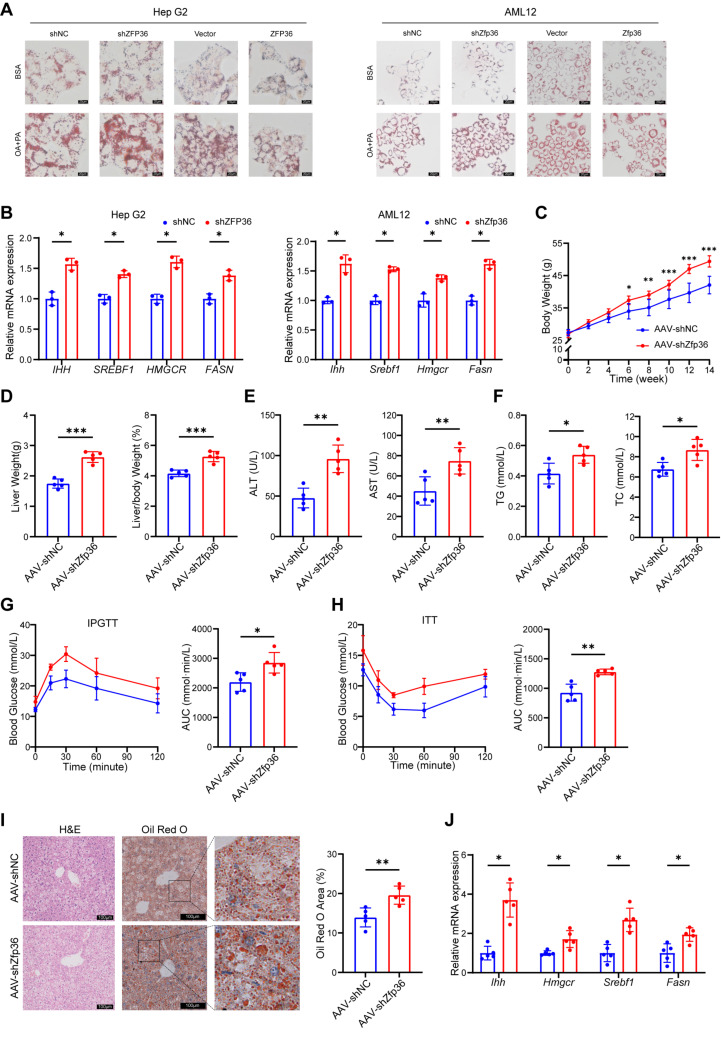
Hepatocellular ZFP36 alleviates MAFLD. (**A**) OA and PA were used to induce lipid accumulation in Hep G2 or AML12 cells in vitro. Cells were transfected with ZFP36 overexpressing or knockdown plasmids. Oil Red O staining of cells. Scale bar, 20 μm. (**B**) mRNA level of *IHH*, *SREBF1*, *HMGCR* and *FASN* in Hep G2 or AML12 cells transfected with shZFP36 or shNC plasmids. (**C**–**J**) Mice injected with AAV-shZfp36 (*n* = 5) or AAV-shNC (*n* = 5) were fed with HFD. (**C**) Mouse weight. (**D**) Liver weight and liver-to-body weight ratio. (**E**) Serum ALT and AST content. (**F**) Serum TG and TC levels. (**G**) Blood glucose concentration at 0, 15, 30, 60 and 120 min after glucose injection and AUC value of IPGTT. (**H**) Blood glucose concentration at 0, 15,30, 60 and 120 min after insulin injection and AUC value of ITT. (**I**) H&E and Oil Red O staining of liver section, with Oil Red O positive area value. Scale bar, 100 μm. Magnification: 40×. (**J**) mRNA levels of *Ihh*, *Hmgcr*, *Srebf1* and *Fasn* in mouse liver. (* *p* < 0.05, ** *p* < 0.01, *** *p* < 0.001).

To investigate the role of ZFP36 in MASLD progression, we performed in vitro experiments by transfecting Hep G2 and AML12 cells with plasmids to overexpress or knock down ZFP36, followed by induction of lipid deposition using oleic acid and palmitic acid. The results showed that Oil Red O staining was attenuated in cells overexpressing ZFP36, whereas staining was enhanced after ZFP36 knockdown (Figure 3A). Also, the expression of a gene related to fibrosis—*IHH*—and genes associated with lipid synthesis—*SREBF1*, *FASN* and *HMGCR*—increased in ZFP36-knockdown cells, and decreased in ZFP36 overexpressing cells (Figure 3B and Figure S3A).

Subsequently, we constructed mice with hepatocyte-specific knockdown of Zfp36 by tail vein injection of AAV8-TBG-shZfp36 or control virus, followed by high-fat diet feeding. Following Zfp36 knockdown, mouse body weight increased significantly, and liver weight, liver-to-body weight ratio, and white adipose tissue mass were higher compared with control mice (Figure 3C,D and Figure S4A). Levels of ALT, AST, TG, and TC in both serum and liver tissues were elevated, indicating that Zfp36 knockdown exacerbated high-fat diet-induced liver injury and lipid metabolism dysfunction (Figure 3E,F and Figure S4B,C). IPGTT and ITT assays demonstrated that glucose tolerance and insulin sensitivity were further impaired in Zfp36-knockdown mice (Figure 3G,H). Histological analysis of liver sections using H&E and Oil Red O staining revealed aggravated hepatic lipid deposition upon Zfp36 knockdown (Figure 3I). Similarly, the expression of genes associated with liver fibrosis and lipid synthesis—*Ihh*, *Srebf1*, *Fasn*, and *Hmgcr*—was further upregulated in AAV-shZfp36 mice, indicating that knockdown of Zfp36 promoted hepatic fibrosis and enhanced lipid synthesis (Figure 3J).

### 2.4. SG Alleviates MASLD by Upregulating ZFP36

**Figure 4:**
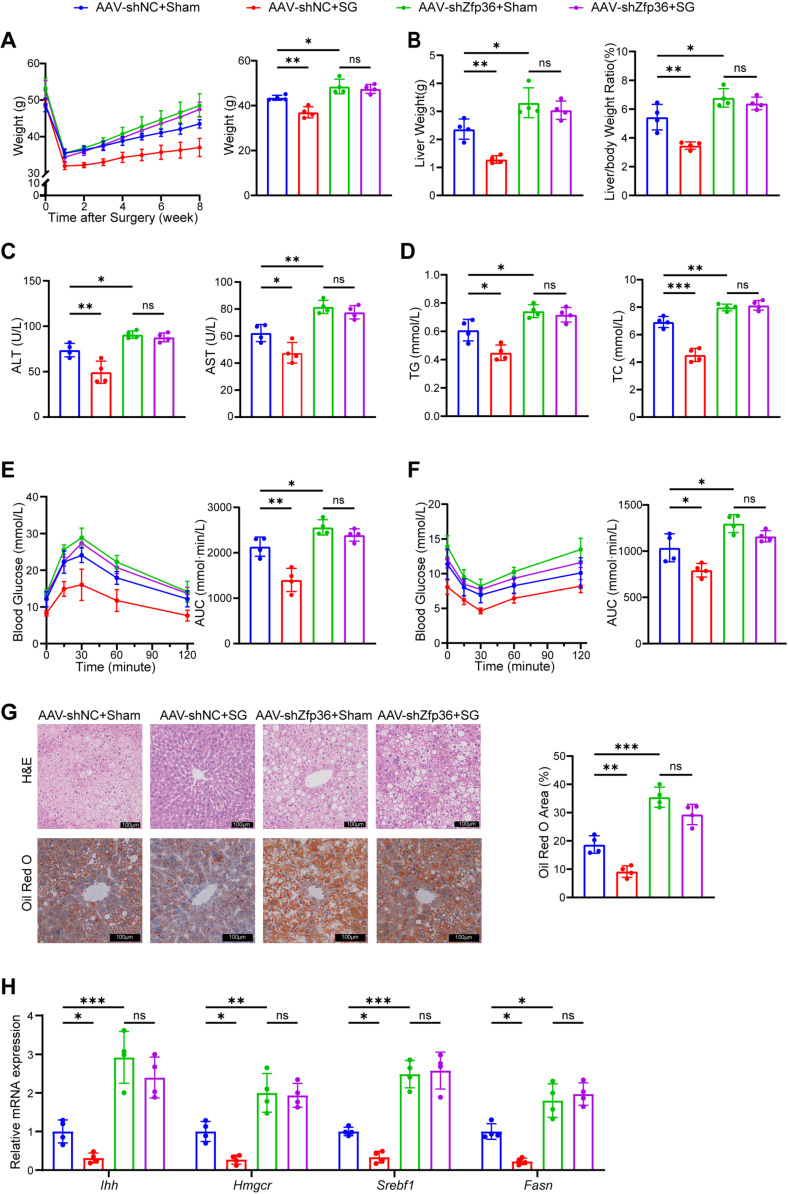
SG alleviates MASLD by upregulating ZFP36. (**A**–**H**) Mice injected with AAV-shZfp36 or AAV-shNC were treated with SG or sham (AAV-shNC+sham, *n* = 4; AAV-shZfp36+sham, *n* = 4; AAV-shNC+SG, *n* = 4; AAV-shZfp36+SG, *n* = 4). (**A**) Mouse weight after surgery every week and the weight at the 8th week. (**B**) Liver weight and liver-to-body weight ratio. (**C**) Serum ALT and AST content. (**D**) Serum TG and TC levels. (**E**) Blood glucose concentration and AUC value of IPGTT. (**F**) Blood glucose concentration and AUC value of ITT. (**G**) H&E and Oil Red O staining of liver sections, with Oil Red O positive area value. Scale bar, 100 μm. (**H**) mRNA levels of *Ihh*, *Hmgcr*, *Srebf1* and *Fasn* in mouse liver. (* *p* < 0.05, ** *p* < 0.01, *** *p* < 0.001).

To determine if ZFP36 directly mediates the reparative process induced by SG against MASLD, we performed sham or SG surgeries on control and hepatocyte-specific Zfp36 knockdown mice. By the eighth week after surgery, control mice treated with SG showed a significant reduction in body weight compared to those receiving sham surgery. In contrast, among Zfp36-knockdown mice, SG and sham surgery had no significant effect on body weight, and both groups exhibited higher body weights than the control mice (Figure 4A). Similarly, SG reduced liver weight, liver-to-body weight ratio, and white adipose tissue mass in control mice, but these effects were not observed in Zfp36-knockdown mice (Figure 4B and Figure S5A). We then measured the levels of transaminases, triglycerides, and cholesterol in the serum and liver tissues (Figure 4C,D and Figure S5B,C). The results indicated that SG treatment lowered the concentrations of these molecular markers, while Zfp36 knockdown inhibited this beneficial effect of SG. IPGTT and ITT assays further demonstrated that Zfp36 knockdown impaired the ability of SG to improve glucose tolerance and insulin sensitivity (Figure 4E,F). Consistent findings were observed at the histological level: the ameliorative effect of SG on hepatic lipid deposition was markedly attenuated upon Zfp36 knockdown (Figure 4G). In addition, the expression of genes associated with fibrosis and lipid synthesis in the liver was significantly elevated following Zfp36 knockdown and remained largely unchanged after either SG or sham surgery (Figure 4H). These results indicate that knockdown of ZFP36 suppresses the ability of SG to mitigate liver injury, hepatic lipid accumulation, and insulin resistance, suggesting that SG exerts its therapeutic effects against MASLD through a mechanism involving ZFP36.

### 2.5. ZFP36 Binds to TEAD4 mRNA and Promotes Its Degradation

**Figure 5:**
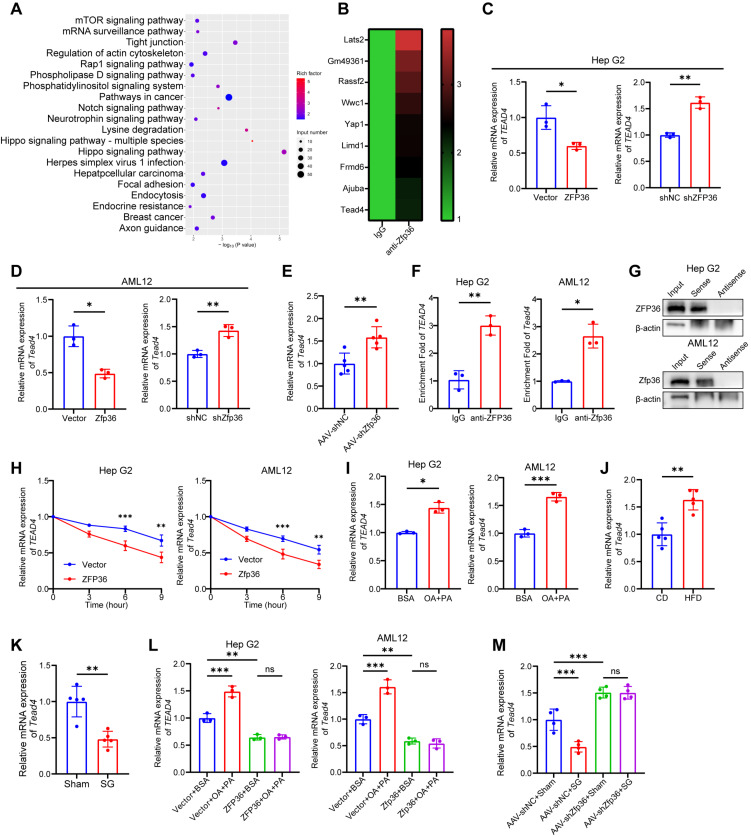
ZFP36 binds to TEAD4 mRNA and promotes its degradation. (**A**) Top 20 KEGG pathways enriched in anti-Zfp36 group using AML12 cells, according to *p*-value. (**B**) mRNAs enriched in Hippo signaling pathway in anti-Zfp36 group, ranked according to fold change. (**C**,**D**) mRNA level of *TEAD4* in Hep G2 or AML12 cells transfected with ZFP36 overexpressing or knockdown plasmids. (**E**) mRNA level of *Tead4* in liver of mice injected with AAV-shZfp36 or AAV-shNC. (**F**) In RIP-qPCR assay, enrichment fold of *TEAD4* in anti-ZFP36 group relative to IgG group in Hep G2 or AML12 cells. (**G**) Protein level of ZFP36 in input, sense and antisense group in RNA pull-down assay in Hep G2 or AML12 cells. (**H**) mRNA level of *TEAD4* in Hep G2 or AML12 cells treated with Act D for different periods of time. (**I**) mRNA level of *TEAD4* in Hep G2 or AML12 cells treated with OA+PA or BSA. (**J**) mRNA level of *TEAD4* in liver of mice fed with CD or HFD. (**K**) mRNA level of *TEAD4* in liver of mice underwent SG or sham. (**L**) mRNA level of *TEAD4* in Hep G2 or AML12 cells transfected with ZFP36 overexpressing or vector plasmids followed by OA+PA or BSA treatment. (**M**) mRNA level of *TEAD4* in liver of mice injected with AAV-shZfp36 or AAV-shNC followed with SG or sham. (* *p* < 0.05, ** *p* < 0.01, *** *p* < 0.001).

ZFP36 has been reported as an RNA-binding protein. We hypothesized that it exerts its inhibitory effect on MASLD through its RNA-binding function. We performed RNA immunoprecipitation followed by RNA sequencing (RIP-seq) using anti-ZFP36 antibody and isotype IgG. mRNAs bound to ZFP36 were significantly enriched in the Hippo signaling pathway (Figure 5A). Though YAP and TAZ are the core proteins of the Hippo pathway, Western blot analysis showed that ZFP36 had no significant effect on the protein levels or phosphorylation status of YAP or TAZ (Figure S6A). Similarly, ZFP36 did not affect the nuclear translocation of YAP or TAZ (Figure S6B). These results suggest that downstream effectors of YAP/TAZ might be downstream of ZFP36. RIP-seq data indicated that ZFP36 could bind to TEA domain transcription factor 4 (TEAD4) mRNA, which is one of the following factors for YAP/TAZ (Figure 5B). RT-qPCR and Western blot analyses revealed that ZFP36 lowered both the mRNA and protein levels of TEAD4 in both in vivo and in vitro settings (Figure 5C–E and Figure S6C,D). RIP assays showed ZFP36 bound to TEAD4 mRNA (Figure 5F). We designed and synthesized a plasmid containing the sense strand of TEAD4 mRNA and a control plasmid containing its antisense sequence. RNA transcribed in vitro from these constructs was used for RNA pull-down experiments to confirm RIP data. The results showed that the TEAD4 sense RNA successfully pulled down the ZFP36 protein, while the antisense RNA failed to pull it down, further confirming their specific interaction (Figure 5G). To assess TEAD4 mRNA degradation, transcription was inhibited with actinomycin D. Overexpression of ZFP36 accelerated the degradation of TEAD4 mRNA (Figure 5H). Those clarified that ZFP36 regulated the Hippo signaling pathway via binding TEAD4 mRNA and reducing its stability.

### 2.6. ZFP36 Alleviates MASLD by Downregulating TEAD4

Next, we investigated the relation of ZFP36 and TEAD4 in MASLD. In vitro, TEAD4 expression was elevated in cell models of lipid deposition induced by oleic acid and palmitic acid (Figure 5I and Figure S7A). Correspondingly, TEAD4 expression was upregulated in the liver tissues of mice fed with a high-fat diet, while it was downregulated in the liver tissues of mice that underwent SG surgery, exhibiting an inverse trend to that of ZFP36 (Figure 5J,K and Figure S7B,C). Furthermore, when cells overexpressing ZFP36 were treated with oleic and palmitic acids, the upregulation of TEAD4 expression by fatty acids was abolished (Figure 5L and Figure S7D). In the in vivo model, knockdown of Zfp36 inhibited the ability of SG treatment to reduce Tead4 expression (Figure 5M and Figure S7E). These results indicate that TEAD4 expression is elevated in MASLD, downregulated after SG treatment, and regulated by ZFP36.

**Figure 6:**
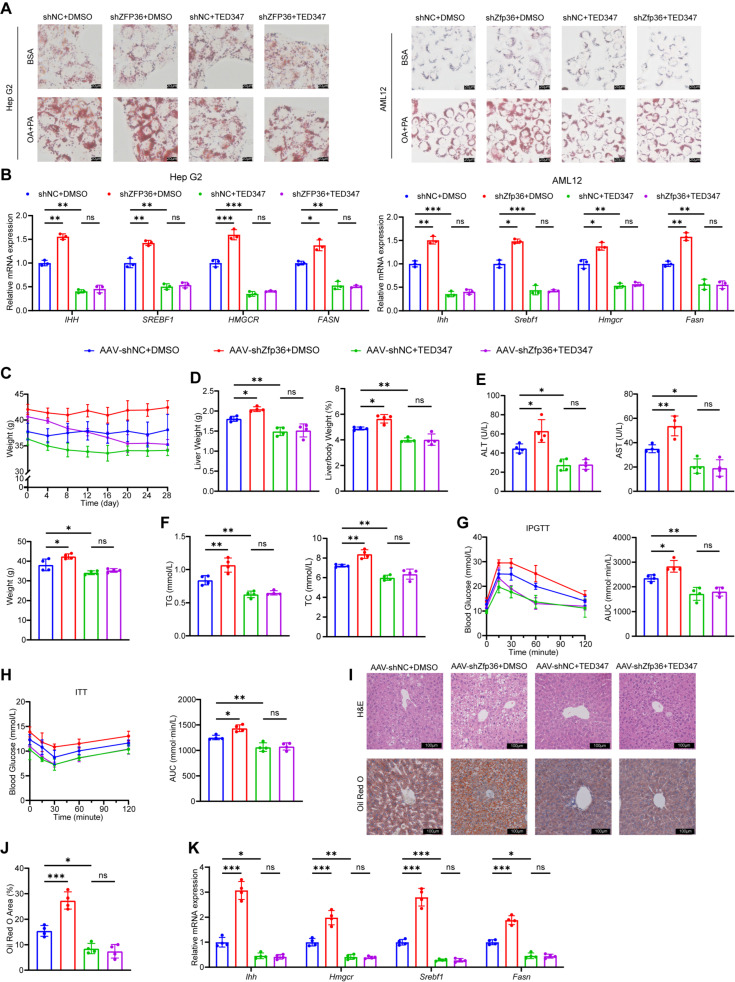
ZFP36 alleviates MASLD by downregulating TEAD4. (**A**) OA and PA were used to induce lipid accumulation in Hep G2 or AML12 cells in vitro. Cells were transfected with shZFP36 or shNC plasmids followed by TED347 or DMSO treatment. Oil Red O staining of cells. Scale bar, 20 μm. (**B**) mRNA level of *IHH*, *SREBF1*, *HMGCR* and *FASN* in Hep G2 or AML12 cells transfected with shZfp36 or shNC plasmids, followed by TED347 or DMSO treatment. (**C**–**J**) Mice injected with AAV-shZfp36 or AAV-shNC were fed with HFD, following TED347 or DMSO intraperitoneal injection (AAV-shNC+DMSO, *n* = 4; AAV-shZfp36+DMSO, *n* = 4; AAV-shNC+TED347, *n* = 4; AAC-shZfp36+TED347, *n* = 4). (**C**) Mouse weight and the weight at the 28th day. (**D**) Liver weight and liver-to-body weight ratio. (**E**) Serum ALT and AST content. (**F**) Serum TG and TC levels. (**G**) Blood glucose concentration and AUC value of IPGTT. (**H**) Blood glucose concentration and AUC value of ITT. (**I**) H&E and Oil Red O staining of liver sections. Scale bar, 100 μm. (**J**) Oil Red O positive area value. (**K**) mRNA levels of *Ihh*, *Hmgcr*, *Srebf1* and *Fasn* in mouse liver. (* *p* < 0.05, ** *p* < 0.01, *** *p* < 0.001).

To determine whether ZFP36 acts through TEAD4, we treated HepG2 and AML12 cells with TED347, a compound that inhibits the transcriptional activity of TEAD4. The results showed that knockdown of Zfp36 exacerbated lipid deposition induced by oleic and palmitic acids, and this effect was significantly suppressed by TED347 treatment (Figure 6A). The expression of genes associated with hepatic fibrosis and lipid synthesis was also inhibited by TED347 (Figure 6B). Subsequently, we administered TED347 or DMSO to Zfp36-knockdown and control mice. By the end of the treatment period, TED347 treatment reduced body weight, with no significant difference observed between Zfp36-knockdown and control mice (Figure 6C). TED347 also attenuated the increases in liver weight, liver-to-body weight ratio, and white adipose tissue mass induced by Zfp36 knockdown (Figure 6D and Fiugure S8A). Levels of transaminases, triglycerides, and cholesterol in serum and liver tissues indicated that TED347 suppressed the exacerbation of liver injury and lipid metabolism dysfunction caused by Zfp36 knockdown (Figure 6E,F and Figure S8B,C). IPGTT and ITT assays similarly demonstrated that TED347 treatment significantly improved glucose tolerance and insulin sensitivity (Figure 6G,H). Histological staining of liver tissue further confirmed that TED347 treatment markedly reduced hepatic lipid deposition (Figure 6I). The expression of fibrosis- and lipid synthesis-related genes in the tissues was also inhibited by TED347 (Figure 6J). These findings indicate that ZFP36 alleviates MASLD by downregulating TEAD4.

### 2.7. ZFP36 Expression Is Negatively Correlated with TEAD4 Expression in MASLD Patients

**Figure 7:**
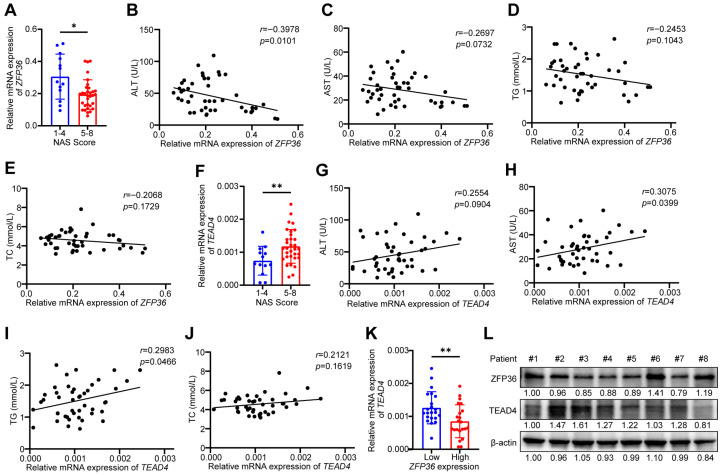
ZFP36 expression is negatively correlated with TEAD4 expression in MASLD patients. (**A**–**K**) mRNA level of *ZFP36* and *TEAD4* level were measured in MASLD patient liver biopsy samples. (**A**) mRNA level of *ZFP36* in NAS 1–4 group (*n* = 13) and 5–8 group (*n* = 32). (**B**) Correlation of mRNA level of *ZFP36* and serum ALT level. (**C**) Correlation of mRNA level of *ZFP36* and serum AST level. (**D**) Correlation of mRNA level of *ZFP36* and serum TG level. (**E**) Correlation of mRNA level of *ZFP36* and serum TC level. (**F**) mRNA level of *TEAD4* in NAS 1–4 group and 5–8 group. (**G**) Correlation of mRNA level of *TEAD4* and serum ALT level. (**H**) Correlation of mRNA level of *TEAD4* and serum AST level. (**I**) Correlation of mRNA level of *TEAD4* and serum TG level. (**J**) Correlation of mRNA level of *TEAD4* and serum TC level. (**K**) mRNA level of *TEAD4* in ZFP36 low-expressing and high-expressing groups, divided according to median expression. (**L**) Protein level of ZFP36 and TEAD4 in patient liver samples. Relative protein level was marked under the. (* *p* < 0.05, ** *p* < 0.01).

We collected liver biopsy samples from 45 MASLD patients and measured the expression levels of ZFP36 and TEAD4. Based on the histopathological NAS, the samples were divided into two groups: a mild-to-moderate group (scores 1–4) and a severe group (scores 5–8). The results showed that ZFP36 expression was lower in the severe group, indicating that ZFP36 expression decreases with MASLD progression (Figure 7A). Furthermore, ZFP36 expression was negatively correlated with serum levels of ALT, AST, triglycerides, and cholesterol in MASLD patients, suggesting that ZFP36 acts as a suppressor in MASLD (Figure 7B–E). Conversely, TEAD4 expression was elevated in the severe group, implying that increased TEAD4 expression is associated with aggravated MASLD (Figure 7F). Similarly, TEAD4 expression showed a positive correlation with serum levels of ALT, AST, triglycerides, and cholesterol (Figure 7G–J). When patients were stratified into low- and high-ZFP36 expression groups based on the median ZFP36 expression level, TEAD4 expression was significantly lower in the high-ZFP36 group (Figure 7K). Western blot analysis also revealed that tissues with higher ZFP36 expression exhibited relatively lower TEAD4 expression (Figure 7L). These data demonstrate that ZFP36 and TEAD4 expression are inversely correlated in MASLD.

## 3. Discussion

Obesity-associated MASLD has become a more prevalent chronic liver disease, with limited targeted agents [23,24,25]. Previous work has shown that hepatic indexes associated with obesity gradually decline following sleeve gastrectomy in humans and mouse models of disease, while the mechanism was unclear [5,26,27,28,29]. Here, we show that SG induces improvements in disease pathogenesis in a process that requires ZFP36. As ZFP36 hepatic-specific knockdown mice do not respond to the surgery and have increased lipid synthesis gene expression, these findings demonstrate that ZFP36 mediates the reparative process induced by SG. These data implicate RNA degradation in the control of the hepatic pathological changes as an important mechanism of SG action.

RNA degradation is a crucial mechanism in the regulation of gene expression, influencing protein synthesis by modulating mRNA abundance [30]. Our research revealed significant suppression of the RNA degradation pathway in the liver tissues of MASLD. And we identified ZFP36 as a suppressor of MASLD. ZFP36 expression was markedly decreased in both MASLD mice and patients, while it was significantly increased after SG. Research on ZFP36 has predominantly focused on its roles in immune regulation: in T cells, it suppresses the expression of cytokines such as TNF, IFN γ, and GM-CSF, thereby restraining excessive immune responses [31]; in macrophages, ZFP36 inhibits IL-27 expression and dampens CD8^+^ T-cell activation, mitigating immune rejection in kidney allografts [32]; and in macrophages stimulated by IFN β, ZFP36 expression is induced, leading to suppression of multiple cytokines and consequently inhibiting necroptosis and inflammatory responses [33]. In the present study, we demonstrate that ZFP36 is a key regulator in metabolism. Hepatocyte-specific knockdown of ZFP36 exacerbated hepatic steatosis in mice. Furthermore, sleeve gastrectomy (SG) exerted its therapeutic effect by upregulating ZFP36 expression, as the amelioration of MASLD by SG was markedly attenuated upon hepatocyte-specific Zfp36 knockdown. Recent studies also indicate that ZFP36 is involved in metabolic regulation. ZFP36 binds to and promotes the degradation of enolase 2 (ENO2), thereby inhibiting vascular endothelial growth factor (VEGF)-mediated angiogenesis [34], and its downregulation impedes fatty acid synthase (FASN) mRNA degradation, leading to abnormal lipid droplet accumulation and contributing to chemotherapy resistance in non-small cell lung cancer [35]. In addition, ZFP36 downregulates ring finger protein (RNF128) expression in adipocytes, which in turn elevates sirtuin 1 (SIRT1) protein levels, promoting lipolysis and suppressing inflammatory cytokine production [36]. Also, bioinformatics analysis revealed that ZFP36 was downregulated in non-alcoholic fatty liver disease with robust diagnostic accuracy [37]. In the present study, we demonstrate that ZFP36 is a key regulator in MASLD. However, the molecular mechanism by which SG upregulates ZFP36 remains to be elucidated and warrants further investigation.

ZFP36 is a well-documented RNA-binding protein that recognizes specific mRNAs and recruits the CCR4-NOT complex to promote deadenylation and subsequent RNA decay [14]. In our research, we identified the Hippo signaling pathway as the downstream of ZFP36 with RIP-seq in MASLD. The Hippo signaling pathway plays a vital role in regulating hepatocyte development and regeneration [19,20]. Growing evidence indicates that aberrant inactivation of the Hippo pathway contributes to MASLD progression and MASLD-associated hepatocellular carcinoma (HCC) [38,39,40]. The core Hippo effectors YAP/TAZ, upon nuclear translocation, must bind to TEAD transcription factors to activate downstream gene expression, highlighting TEADs as critical regulators of Hippo-mediated transcription. For instance, the E3 ubiquitin ligase ring finger protein 214 (RNF214) mediates non-degradative ubiquitination of TEAD1-4, enhancing TEAD–YAP interaction and promoting HCC progression, while knockout of Tead2 and Tead4 markedly suppresses TAZ-driven HCC proliferation [41]. In MASLD livers, elevated TEAD1 expression promotes lipid deposition by activating pathways related to fatty acid synthesis, cholesterol synthesis, and AMPK signaling [21]. In this study, we found that ZFP36 modulates the Hippo pathway primarily by affecting TEAD4 expression, rather than through YAP/TAZ. Inhibition of TEAD4 transcriptional activity significantly alleviated the aggravated lipid accumulation induced by ZFP36 knockdown.

**Figure 8:**
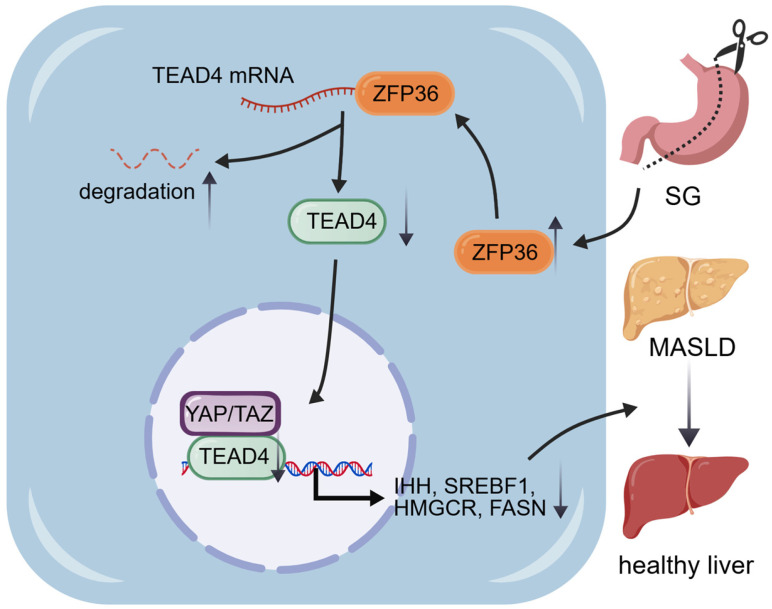
Graphic abstract of sleeve gastrectomy ameliorating MASLD through upregulating ZFP36 expression. ZFP36 was upregulated after sleeve gastrectomy in hepatocytes, which binds to *TEAD4* mRNA and promotes its degradation. Reduced TEAD4 inhibited hippo signaling pathway activity, leading to decreased transcription of IHH, SREBF1, HMGCR and FASN, and improvement of MASLD. This figure was created with BioGDP (http://biogdp.com) [42].

In summary, this study reveals that sleeve gastrectomy alleviates MASLD by upregulating ZFP36 expression in hepatocytes, which promotes the degradation of TEAD4 mRNA, thereby suppressing the expression of lipid synthesis-related genes and ultimately improving hepatic steatosis, insulin resistance, and lipid metabolism disorders. Our study unveils a novel mechanism by which SG ameliorates MASLD via ZFP36, and suggests that activating ZFP36 may represent a promising new therapeutic approach for MASLD, thereby expanding the available treatment strategies (Figure 8).

## 4. Materials and Methods

### 4.1. Animals

Six-week-old male C57BL/6J wild-type mice, purchased from Vital River Laboratory Animal Technology Co., Ltd. (Beijing, China), were fed with high-fat diet (HFD) or chow diet (CD) for 16 weeks. Body weight was measured every week. A total of 10 mice were fed with CD (*n* = 5) and HFD (*n* = 5). All mice were raised in an SPF environment at the Model Animal Research Center of Shandong University.

To conduct hepatocyte-specific knockdown Zfp36 mice, we applied adeno-associated virus serotype 8 (AAV8), namely AAV8-TBG-shZfp36 and its negative control AAV. The virus was injected through tail vein into 6-week-old male C57BL/6J mice at the dose of 6 × 10^11^ vg per mice. After being fed a chow diet for 2 weeks, the mice were then fed with HFD for 16 weeks. A total of 10 mice were injected with AAV-shNC (*n* = 5) and AAV-shZfp36 (*n* = 5).

After 16-week HFD, the mice (weight > 42 g) were randomly assigned into two groups: sham or SG. SG procedure involves anesthesia with isoflurane, opening the abdominal cavity, dissociating the stomach from surrounding tissues and achieving hemostasis, resecting approximately 80% of the gastric volume along the greater curvature, suturing the gastric wall, and closing the abdominal cavity. For the sham group, the stomach was mobilized, followed by an incision and subsequent suturing of the gastric wall, after which the abdominal cavity was closed. Meloxicam was injected subcutaneously to relieve pain. Mice were fed with HFD for 8 weeks after the surgery. A total of 12 mice fed with HFD were treated with sham (*n* = 5) and SG (*n* = 7). Two SG mice died within one week after operation. Then, a total of 24 mice were injected with AAV-shNC (*n* = 12) and AAV-shZfp36 (*n* = 12). After being fed with HFD, mice were randomly assigned to undergo sham or SG operation, 6 mice for each group (AAV-shNC+sham, AAV-shZfp36+sham, AAV-shNC+SG and AAV-shZfp36+SG). One mouse in AAV-shNC+sham group, two mice in AAV-shNC+SG and one mouse in AAV-shZfp36+SG group died after surgery. We selected 4 mice from each group for data analysis.

After 16 weeks of HFD feeding, the mice were randomly assigned to two groups, receiving TED347 and DMSO treatment, respectively. TED347 was administered via intraperitoneal injection at a dose of 20 mg/kg once every 4 days, 28 days in total. A total of 20 mice were injected with AAV-shNC (*n* = 10) and AAV-shZfp36 (*n* = 10). After being fed with HFD for 16 weeks, mice were randomly assigned to receive either DMSO or TED347 treatment, 5 mice for each group (AAV-shNC+DMSO, AAV-shZfp36+DMSO, AAV-shNC+TED347 and AAV-shZfp36+TED347). Finally, we randomly selected 4 mice from each group for data analysis.

The animal research was approved by the Laboratory Animal Ethical and Welfare Committee of Shandong University Cheeloo College of Medicine (Approval No. 23095).

The detailed information of chemicals and kits used for animal experiments are listed in Tables S3 and S4.

### 4.2. Intraperitoneal Glucose Tolerance Test (IPGTT) and Insulin Tolerance Test (ITT)

After a 12 h fast, blood glucose levels were measured from the tail vein. Subsequently, glucose (2 g/kg) or insulin (0.75 U/kg) was administered via intraperitoneal injection, and tail vein blood glucose was measured at 15, 30, 60, and 120 min post-injection.

### 4.3. Cell Culture

Hep G2 cells were cultured in MEM containing NEAA and 10% FBS (Procell, Wuhan, China). AML12 cells were cultured in AML12 Cell Complete Medium (Procell, Wuhan, China). Both cell lines were purchased from National Collection of Authenticated Cell Cultures (Shanghai, China). Cells were cultured at 37 °C with 5% CO_2_.

To induce lipid accumulation in vitro, oleic acid (0.2 mM) and palmitic acid (0.1 mM) were added to culture medium and incubated for 24 h. Isopyknic 12% BSA was used as negative control.

Plasmids used for cell transfection were acquired from Research Cloud Biology (Jinan, China). Plasmids for overexpression of ZFP36 were constructed on pcDNA 3.1 vector. shRNA of human *ZFP36* (sequence: GACGGAACTCTGTCACAAGTT) and shRNA of mouse *Zfp36* (sequence: ACCACCTCCTCTCGATACAAG) were constructed on pLKO.1 vector.

### 4.4. Real-Time Quantitative Polymerase Chain Reaction

Total RNA was isolated using RNA-easy Isolation Reagent from cells and liver tissues, and reverse transcribed to cDNA using Evo M-MLV RT Premix (Accurate Biology, Changsha, China) for qPCR, followed by real-time quantitative polymerase chain reaction (RT-qPCR) analysis on Bio-Rad Company (Hercules, CA, USA) equipment using SYBR Green Premix Pro Taq HS qPCR Kit (Accurate Biology, Changsha, China).

Primers used for RT-qPCR are shown in Table S1.

### 4.5. RNA Immunoprecipitation, Library Preparation and Sequencing

RNA immunoprecipitation was performed using PureBinding RNA Immunoprecipitation Kit (Geneseed, Guangzhou, China) according to manufacturer’s protocol. In brief, following the incubation of anti-ZFP36 antibody or IgG with magnetic beads, the cell lysate containing proteins and RNA was added for co-incubation. The beads were then collected, and the bound RNA was eluted for subsequent RNA sequencing or qPCR analysis.

For high-throughput RNA sequencing, libraries were prepared using KCTM Digital Stranded mRNA Library Prep Kit (Seqhealth Tech. Co., Ltd., Wuhan, China) following the manufacturer’s instructions. The kit eliminates duplication bias and errors in PCR and sequencing steps, by using a unique molecular identifier (UMI) of 12 random bases

### 4.6. RNA Pull-Down

TEAD4 mRNA and control RNA were synthesized using the MEGAscript T7 Transcription Kit (Thermo Fisher, Waltham, MA, USA) along with biotin-UTP. After purification with Dynabeads RNA Purification Kit (Thermo Fisher, Waltham, MA, USA), 1 μg RNA was incubated with 3 μg total cellular protein and streptavidin-conjugated magnetic beads. Following protein elution, a Western blot was performed to detect proteins bound to the TEAD4 mRNA.

### 4.7. Western Blot

Total protein was extracted using RIPA buffer added with proteasome inhibitor cocktail from cells and liver tissues. BCA Protein Assay Kit (NCM Biotechnology, Suzhou, China) was used to measure protein concentration. After being denatured at 100 °C for 5 min with SDS-PAGE loading buffer, proteins were resolved by SDS-PAGE, followed by electro-transferred to PVDF membranes. After blocking with skim milk, the membranes were incubated with primary antibodies at 4 °C overnight. The primary antibodies used are shown in Supplementary Table S2. The membranes were incubated with Goat Anti-Rabbit IgG (H+L) HRP or Goat Anti-Mouse IgG (H+L) HRP for 1 h at room temperature. Protein bands were visualized using supersensitive ECL chemiluminescent solution and a MyECL Imager (Qinxiang, Shanghai, China).

### 4.8. Cytoplasmic-Nuclear Protein Separation

Cytoplasmic and nuclear proteins were separated using a Nuclear and Cytoplasmic Protein Extraction Kit (Beyotime Biotechnology, Shanghai, China) according to the manufacturer’s protocol. A Western blot was performed to determine the protein expression levels in the isolated cytoplasmic and nuclear fractions.

### 4.9. Actinomycin D Chase Assay

After being transfected with ZFP36 or vector plasmids for 48 h, cells were treated with actinomycin D (5 μg/mL), and collected at 0 h, 3 h, 6 h and 9 h. RT-qPCR was used to measure the stability of TEAD4 mRNA.

### 4.10. Human Tissue Specimens

A total of 45 fresh liver biopsy samples were collected from obesity patients undergoing bariatric surgery at the Department of General Surgery, Qilu Hospital of Shandong University (Jinan, China). All liver biopsy specimens were histologically confirmed as MASLD, with cases of alcoholic hepatitis and viral hepatitis excluded. Liver tissues from patients with malignant tumors, hyperthyroidism, hypothyroidism, or type 2 diabetes were also excluded. All liver tissue samples were snap-frozen in liquid nitrogen and stored. Histological evaluation and NASH Activity Score (NAS) scoring was performed by two pathologists independently. All samples used in this study were obtained with patients’ informed consent. The research protocols were reviewed and approved by the Ethical Committee of Qilu Hospital of Shandong University (Approval No. KYLL-2017-073).

### 4.11. Statistical Analysis

All statistical analyses were performed using GraphPad Prism 9.0 (GraphPad Software, San Diego, CA, USA). The experimental results are expressed as mean ± standard deviation (SD). Comparisons between two groups were performed using an unpaired Student’s *t*-test. Differences among multiple groups were analyzed by analysis of variance (ANOVA). Correlations between the expression levels of ZFP36 and TEAD4 and the contents of ALT, AST, TG, and TC were assessed using Pearson correlation analysis. A two-tailed *p*-value < 0.05 was considered statistically significant (* *p* < 0.05, ** *p* < 0.01, *** *p* < 0.001).

## Data Availability

The original contributions presented in this study are included in the article/Supplementary Material. Further inquiries can be directed to the corresponding author.

## Supplementary Materials

The following supporting information can be downloaded at: https://www.mdpi.com/article/10.3390/ijms27093736/s1.

## Abbreviations

The following abbreviations are used in this manuscript:

ZFP36	Zinc finger protein 36
TEAD4	TEA domain transcription factor 4
YAP1	Yes-associated protein 1
TAZ	WW-domain-containing transcription regulator 1
MASLD	Metabolic dysfunction-associated steatotic liver disease
SG	Sleeve gastrectomy
OA	Oleic acid
PA	Palmitic
HFD	High-fat diet
WAT	White adipose tissue
ALT	Alanine aminotransferase
AST	Aspartate aminotransferase
TG	triglyceride
TC	Total cholesterol
IPGTT	Intraperitoneal glucose tolerance test
ITT	Insulin tolerance test
H&E	Hematoxylin and eosin
ORO	Oil Red O
IgG	Immunoglobulin G
RIP	RNA immunoprecipitation
