# Supplementary material for: ZFP36 Alleviates MASLD Through Facilitating TEAD4 mRNA Degradation After Sleeve Gastrectomy
Source: Int J Mol Sci. 2026 Apr 22;27(9):3736. doi: 10.3390/ijms27093736 (PMC13164400; doi:10.3390/ijms27093736)
Supplement: Supplementary file 1 [file ijms-27-03736-s001.zip › ijms-4226460-supplementary.pdf]

Supplementary Figures

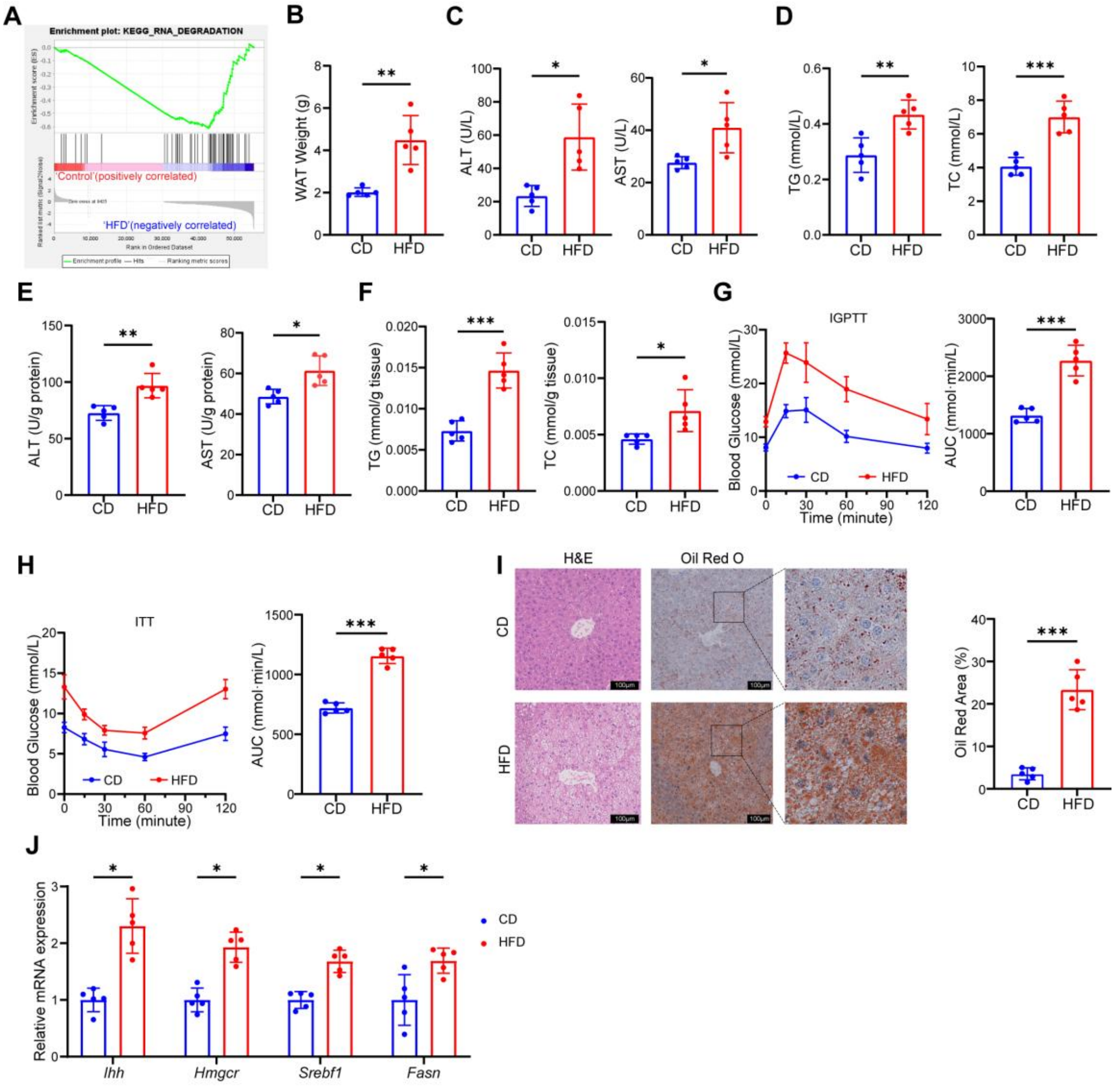

Figure S1. High-fat diet induced MASLD in mice. (A) GSEA analysis of RNA degradation pathway in GSE246211. (B-J) six-week-old male mice fed with CD or HFD. (B) White adipose tissue (WAT) weight. (C) Serum ALT and AST content. (D) Serum TG and TC level. (E) Hepatic ALT and AST content. (F) Hepatic TG and TC level. (G) Blood glucose concentration and AUC value of IPGTT. (H) Blood glucose concentration and AUC value of ITT. (I) H&E and oil red O staining of liver section, with oil red O positive area value. Scale bar, 100 μm. (J) mRNA level of *Ihh*, *Hmgcr*, *Srebf1* and *Fasn* in mice liver. (* $p<0.05$, ** $p<0.01$, *** $p<0.001$)

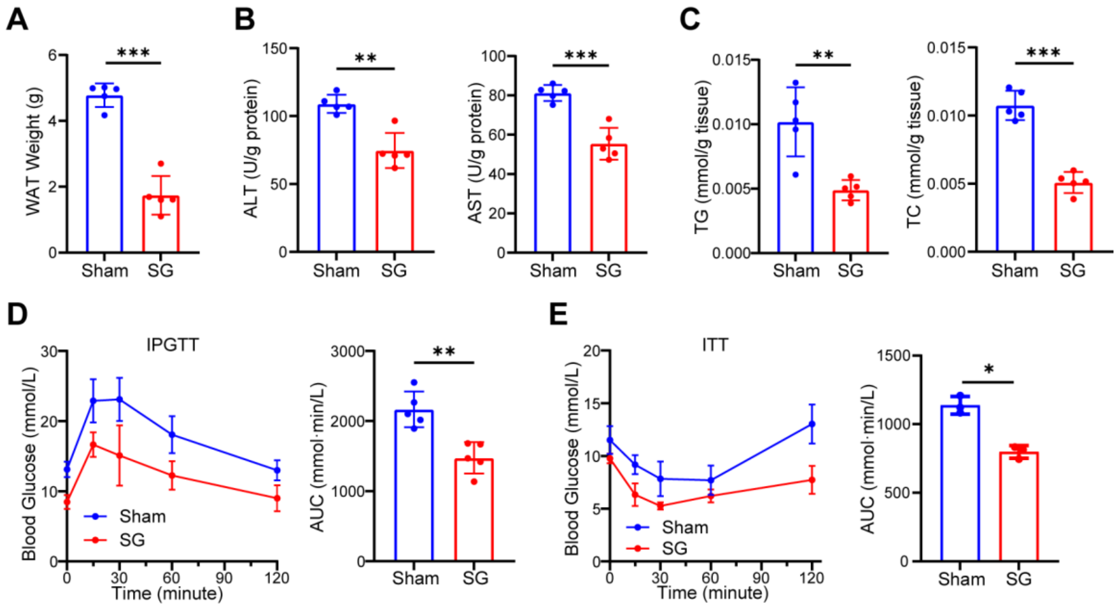

Figure S2. Sleeve gastrectomy ameliorates MASLD. (A-E) HFD mice underwent sleeve gastrectomy (SG) or sham treatment. (A) WAT weight. (B) Hepatic ALT and AST content. (C) Hepatic TG and TC level. (D) Blood glucose concentration and AUC value of IPGTT. (E) Blood glucose concentration and AUC value of ITT. (* p<0.05, ** p<0.01, *** p<0.001)

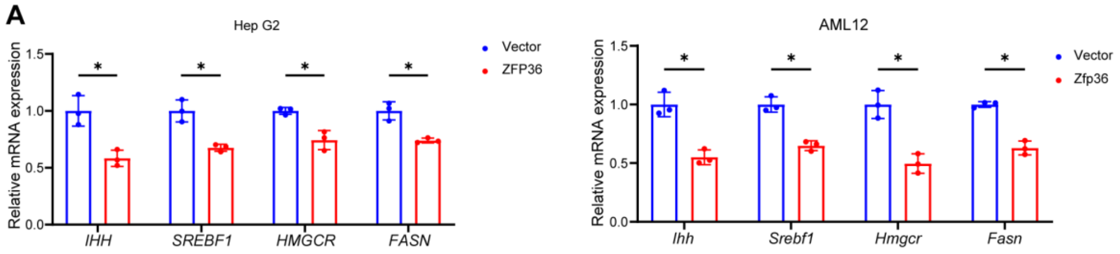

Figure S3. ZFP36 inhibits the expression of genes related to fibrosis and lipid synthesis. (A) mRNA level of *IHH*, *SREBF1*, *HMGCR* and *FASN* in Hep G2 or AML12 cells transfected with ZFP36 overexpressing or control plasmids. (* p<0.05, ** p<0.01, *** p<0.001)

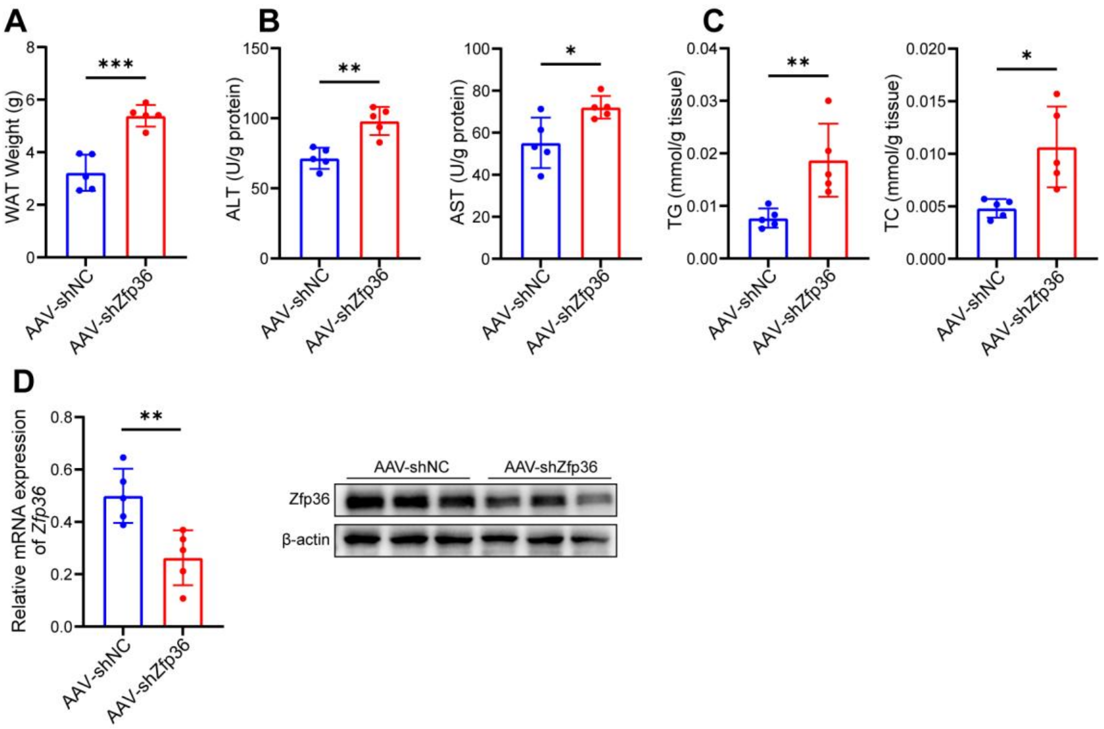

Figure S4. Hepatocyte-specific knockdown of Zfp36 exacerbates MASLD. (A-D) Mice injected with AAV-shZfp36 or AAV-shNC were fed with HFD. (A) WAT weight. (B) Hepatic ALT and AST content. (C) Hepatic TG and TC level. (D) mRNA and protein level in mice liver. (* $p<0.05$, ** $p<0.01$, *** $p<0.001$)

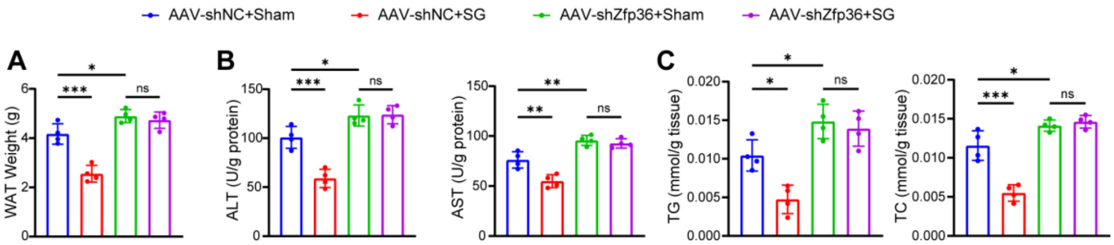

Figure S5. SG alleviates MASLD by upregulating ZFP36. (A-C) Mice injected with AAV-shZfp36 or AAV-shNC were treated with SG or sham. (A) WAT weight. (B) Hepatic ALT and AST content. (C) Hepatic TG and TC level. (* $p<0.05$, ** $p<0.01$, *** $p<0.001$)

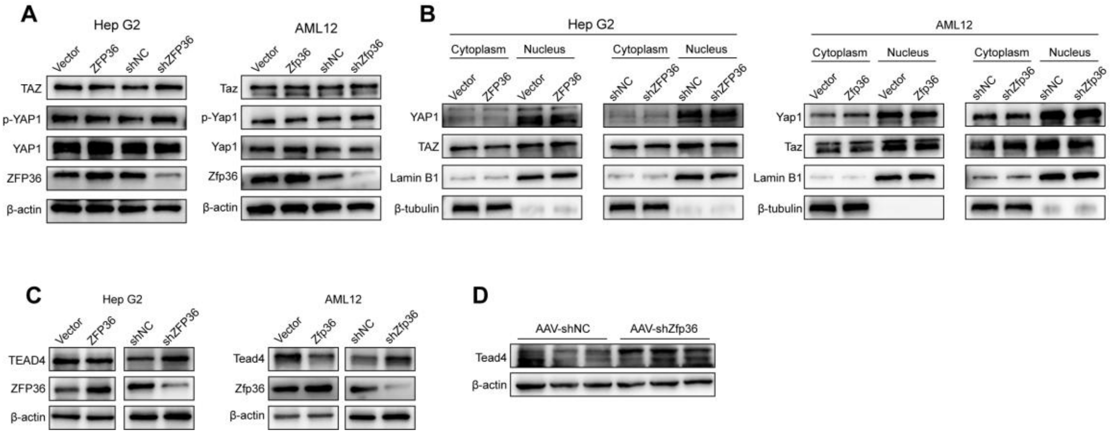

Figure S6. ZFP36 regulates hippo pathway by downregulating TEAD4 expression rather than YAP/TAZ. (A) Protein level of YAP1, p-YAP1 and TAZ in Hep G2 or AML12 cells transfected with ZFP36 overexpressing or knockdown plasmids. (B) Protein level of YAP1 and TAZ in cytoplasm and nucleus in Hep G2 or AML12 cells transfected with ZFP36 overexpressing or knockdown plasmids. (C) Protein level of TEAD4 in Hep G2 or AML12 cells transfected with ZFP36 overexpressing or knockdown plasmids. (D) Protein level of TEAD4 in liver of mice injected with AAV-shZfp36 or shNC followed with HFD. (* p<0.05, ** p<0.01, *** p<0.001)

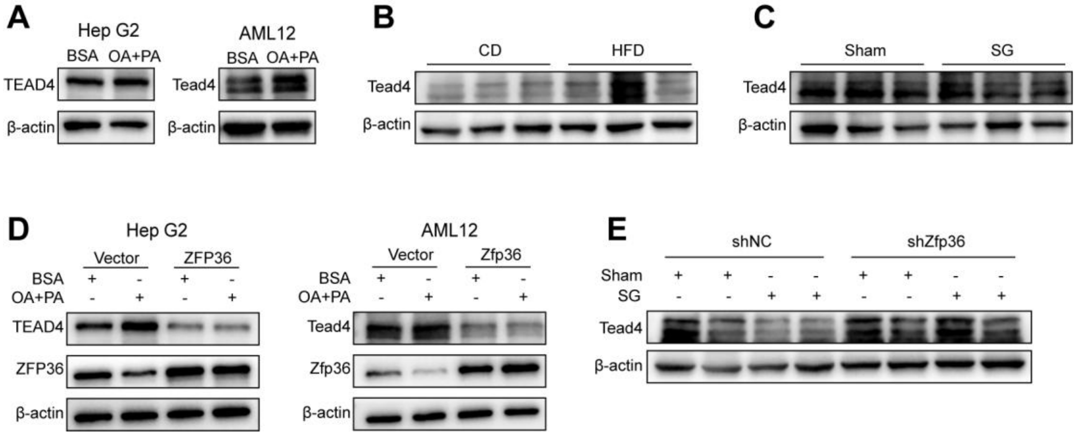

Figure S7. The upregulation of TEAD4 expression in MASLD is regulated by ZFP36. (A) Protein level of TEAD4 in cells treated with OA+PA or BSA. (B) Protein level of TEAD4 in liver of mice fed with CD or HFD. (C) Protein level of TEAD4 in liver of mice underwent SG or sham. (D) Protein level of TEAD4 in cells transfected with ZFP36 overexpressing or vector plasmids followed with OA+PA or BSA treatment. (E) Protein level of TEAD4 in liver of mice injected AAV-shZfp36 or AAV-shNC followed with SG or sham. (* p<0.05, ** p<0.01, *** p<0.001)

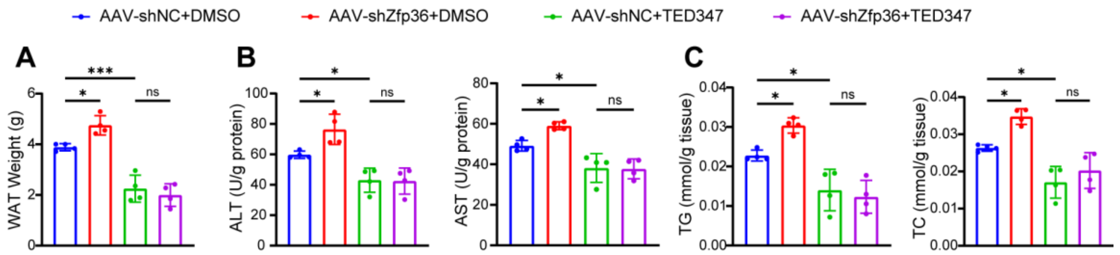

Figure S8. ZFP36 alleviates MASLD by downregulating TEAD4. (A-C) Mice injected with AAV-shZfp36 or AAV-shNC were fed with HFD, following TED347 or DMSO intraperitoneal injection. (A) WAT weight. (B) Hepatic ALT and AST content. (C) Hepatic TG and TC level. (* p<0.05, ** p<0.01, *** p<0.001)

Table S1. Primers for RT-qPCR

Primers	Sequence (5'→3')
Homo-ZFP36-F	GACTGAGCTATGTCGGACCTT
Homo-ZFP36-R	GAGTTCCGTCTTGTATTTGGGG
Homo-β-actin-F	TCCTGTGGCATCCACGAA
Homo-β-actin-R	TCGTCATACTCCTGCTTGC
Homo-TEAD4-F	GAACGGGGACCCTCCAATG
Homo-TEAD4-R	GCGAGCATACTCTGTCTCAAC
Homo-IHH-F	AACTCGCTGGCTATCTCGGT
Homo-IHH-R	GCCCTCATAATGCAGGGACT
Homo-SREBF1-F	ACAGTGACTTCCCTGGCCTAT
Homo-SREBF1-R	GCATGGACGGGTACATCTTCAA
Homo-FASN-F	AAGGACCTGTCTAGGTTTGATGC
Homo-FASN-R	TGGCTTCATAGGTGACTTCCA
Homo-HMGCR-F	TGATTGACCTTTCCAGAGCAAG
Homo-HMGCR-R	CTAAAATTGCCATTCCACGAGC
Mus-Zfp36-F	AACGGAACTCTGCCACAAGT
Mus-Zfp36-R	AGTGGCATCGAGAGCCATAG
Mus-β-actin-F	CATTGCTGACAGGATGCAGAAGG

Mus-β-actin-R	TGCTGGAAGGTGGACAGTGAGG
Mus-Tead4-F	CAACCTGGAACATCCCACGAT
Mus-Tead4-R	GAAAGCCGAGAACTCCAACAT
Mus-Ihh-F	CTCTTGCCTACAAGCAGTTCA
Mus-Ihh-R	CCGTGTTCTCCTCGTCCTT
Mus-Srebf1-F	TGACCCGGCTATTCCGTGA
Mus-Srebf1-R	CTGGGCTGAGCAATACAGTTC
Mus-Fasn-F	GGAGGTGGTGATAGCCGGTAT
Mus-Fasn-R	TGGGTAATCCATAGAGCCCAG
Mus-Hmgcr-F	AGCTTGCCCGAATTGTATGTG
Mus-Hmgcr-R	TCTGTTGTGAACCATGTGACTTC

Table S2. Antibodies for western blot

Antibody	Source	Identifier
ZFP36	Abmart	MG172383
β-actin	Abways	AB0035
TEAD4	Abmart	TD13283
YAP1	Proteintech	66900-1-Ig
p-YAP1	Cell Signaling Technology	13008
TAZ	Proteintech	66500-1-Ig
Lamin B1	Abways	AB0054
β-tubulin	Abways	AB0039
Goat Anti-Rabbit IgG (HRP)	Abways	AB0101
Goat Anti-Mouse IgG (HRP)	Abways	AB0102

Table S3. Chemicals

Chemicals	Source	Identifier
HFD	Huafukang	Cat#D12492
Sodium Oleate	Sigma-Aldrich	CAS#143-19-1
Sodium Palmitate	Sigma-Aldrich	CAS#408-35-5
BSA	Sigma-Aldrich	CAS#9048-46-8
Actinomycin D	MedChemExpress	Cat#HY-17559
D-Glucose	Solarbio	CAS#50-99-7
Insulin	Aladdin	CAS#12584-58-6
DMSO	MedChemExpress	Cat#HY-Y0320C
TED347	MedChemExpress	Cat#HY-125269
Oil red O	Solarbio	Cat#G1260
Tween 80	MedChemExpress	Cat#HY-Y1891
PEG 300	MedChemExpress	Cat#HY-Y0873
Bio-16-UTP	Thermo Fisher Scientific	Cat#AM8452

Table S4. Commercial Assays

Critical commercial assays	Source	Identifier
Alanine aminotransferase Assay Kit	Nanjing Jiancheng Bioengineering Institue	C009-2-1
Aspartate aminotransferase Assay Kit	Nanjing Jiancheng Bioengineering Institue	C010-2-1
Triglyceride assay kit	Nanjing Jiancheng Bioengineering Institue	A110-1-1
Total cholesterol assay kit	Nanjing Jiancheng Bioengineering Institue	A111-1-1
Nuclear and Cytoplasmic Protein Extraction Kit	Beyotime	P0027
RNA immunoprecipitation kit	Geneseed	P0102

MEGAscript T7 Transcription Kit	Thermo Fisher Scientific	AM1333
MEGAclear Purification Kit	Thermo Fisher Scientific	AM1908
Dynabead kilobaseBINDER Kit	Thermo Fisher Scientific	60101
